# Long-term exposure to outdoor air pollution and risk factors for cardiovascular disease within a cohort of older men in Perth

**DOI:** 10.1371/journal.pone.0248931

**Published:** 2021-03-29

**Authors:** Stephen Vander Hoorn, Kevin Murray, Lee Nedkoff, Graeme J. Hankey, Leon Flicker, Bu B. Yeap, Osvaldo P. Almeida, Paul Norman, Bert Brunekreef, Mark Nieuwenhuijsen, Jane Heyworth

**Affiliations:** 1 School of Population and Global Health, The University of Western Australia, Perth, Australia; 2 Centre for Air Pollution, Energy and Health Research, Glebe, New South Wales, Australia; 3 Medical School, The University of Western Australia, Crawley, Australia; 4 WA Centre for Health & Ageing, The University of Western Australia, Crawley, Australia; 5 Department of Endocrinology and Diabetes, Fiona Stanley Hospital, Perth, Western Australia, Australia; 6 Institute for Risk Assessment Sciences, Utrecht University, Utrecht, Netherlands; 7 Institute for Global Health, Barcelona, Spain; Universidade Federal do Rio Grande - FURG, BRAZIL

## Abstract

While there is clear evidence that high levels of pollution are associated with increased all-cause mortality and cardiovascular mortality and morbidity, the biological mechanisms that would explain this association are less understood. We examined the association between long-term exposure to air pollutants and risk factors associated with cardiovascular disease. Air pollutant concentrations were estimated at place of residence for cohort members in the Western Australian Centre for Health and Ageing Health in Men Study. Blood samples and blood pressure measures were taken for a cohort of 4249 men aged 70 years and above between 2001 and 2004. We examined the association between 1-year average pollutant concentrations with blood pressure, cholesterol, triglycerides, C-reactive protein, and total homocysteine. Linear regression analyses were carried out, with adjustment for confounding, as well as an assessment of potential effect modification. The four pollutants examined were fine particulate matter, black carbon (BC), nitrogen dioxide, and nitrogen oxides. We found that a 2.25 μg/m^3^ higher exposure to fine particulate matter was associated with a 1.1 percent lower high-density cholesterol (95% confidence interval: -2.4 to 0.1) and 4.0 percent higher serum triglycerides (95% confidence interval: 1.5 to 6.6). Effect modification of these associations by diabetes history was apparent. We found no evidence of an association between any of the remaining risk factors or biomarkers with measures of outdoor air pollution. These findings indicate that long-term PM_2.5_ exposure is associated with elevated serum triglycerides and decreased HDL cholesterol. This requires further investigation to determine the reasons for this association.

## Introduction

Previous epidemiological studies have reported an association between exposure to ambient air pollution and disease of the lungs and cardiovascular system [1–3] and has been estimated to contribute to 3.4 million premature deaths and more than 90 million DALYs each year worldwide [4]. Furthermore, air pollution has emerged as a major contributor to global stroke burden and reducing exposure has been put forward as one of the main priorities to reduce stroke burden [5]. Although toxicological studies have explored possible underlying mechanisms, supportive epidemiological evidence remains scarce.

Two large population-based health surveys have examined the association between air pollution and risk factors for cardiovascular disease (CVD) [6, 7]. Both studies reported a significant positive association between chronic PM_10_ exposure and increased serum concentration of triglycerides. Some studies have reported data supporting a link between exposure to particulate matter (PM) and decreased high-density lipoprotein (HDL) [8, 9]. Epidemiological findings involving high blood pressure and high cholesterol are limited by small sample size and are inconclusive [10–13]. Several studies have observed acute changes to blood markers for inflammation in response to short-term fluctuations in air pollution [14–16]. whereas data on long-term exposure and CVD are more limited [17–20]. Although there is ongoing debate as to whether high sensitivity C-reactive protein (hs-CRP) is an independent predictor of CVD [21, 22], the role of inflammation in the context of air pollution is of considerable interest [23] and determining which CVD-related pathways might be involved remains an important priority for air pollution research. Studies have shown that elevated levels of total homocysteine (tHcy) are a risk factor for cardiovascular disease [24].

Very few studies have been conducted in populations with relatively low ambient air concentrations and therefore data on associations with CVD at the lower end of the pollution range is very limited. This study aimed to investigate the external validity and generalizability of reported associations between long-term exposure to air pollution and recognized risk factors for CVD including blood pressure, cholesterol, and triglycerides as well as inflammatory biomarkers in a cohort of older men in metropolitan Perth, Western Australia.

## Materials and methods

### Study population

Analyses presented in this article are based on data from the Health in Men Study (HIMS), which is a population-based cohort study of older men conducted in Perth, Western Australia. Details regarding the enrolment of participants have been described elsewhere [25]. In brief, 12203 men aged 65 years or older were recruited by random sampling from the Australian electoral roll between 1996 and 1998. All participants from the cohort completed physical and health assessments, and self-reported questionnaires at baseline and at various intervals during follow-up, and have also been followed up using linked hospitalization and mortality data from the Western Australia Data Linkage System [25].

During the years 2001–2004, those men who were still alive were contacted and invited for a follow-up assessment and asked to provide a fasting blood sample (n = 4275). This article refers to those men who attended the follow-up assessment and had a venous blood sample taken.

Participants who lived outside the Metropolitan Perth region, or had missing data on the key baseline variables (education level, history of smoking, daily tobacco consumption, weight, height, and history of CVD) were excluded from analyses. The study was approved by the Human Research Ethics Committee of the University of Western Australia and the Health Department of Western Australia.

### Air pollution exposure variables

Four air pollutants were analyzed in this study. Particulate matter ≤2.5μm in diameter (PM_2.5_), nitrogen dioxide (NO_2_), and nitrogen oxides (NO_x_) were selected as there is substantial evidence of their association with CVD [4]. There is a relatively small body of literature showing the health risks associated with BC, however, it has been hypothesized that black carbon (BC) acts as a carrier of combustion from traffic and may be more useful for capturing exhibits higher spatial variability within area areas [26].

**Fig 1:**
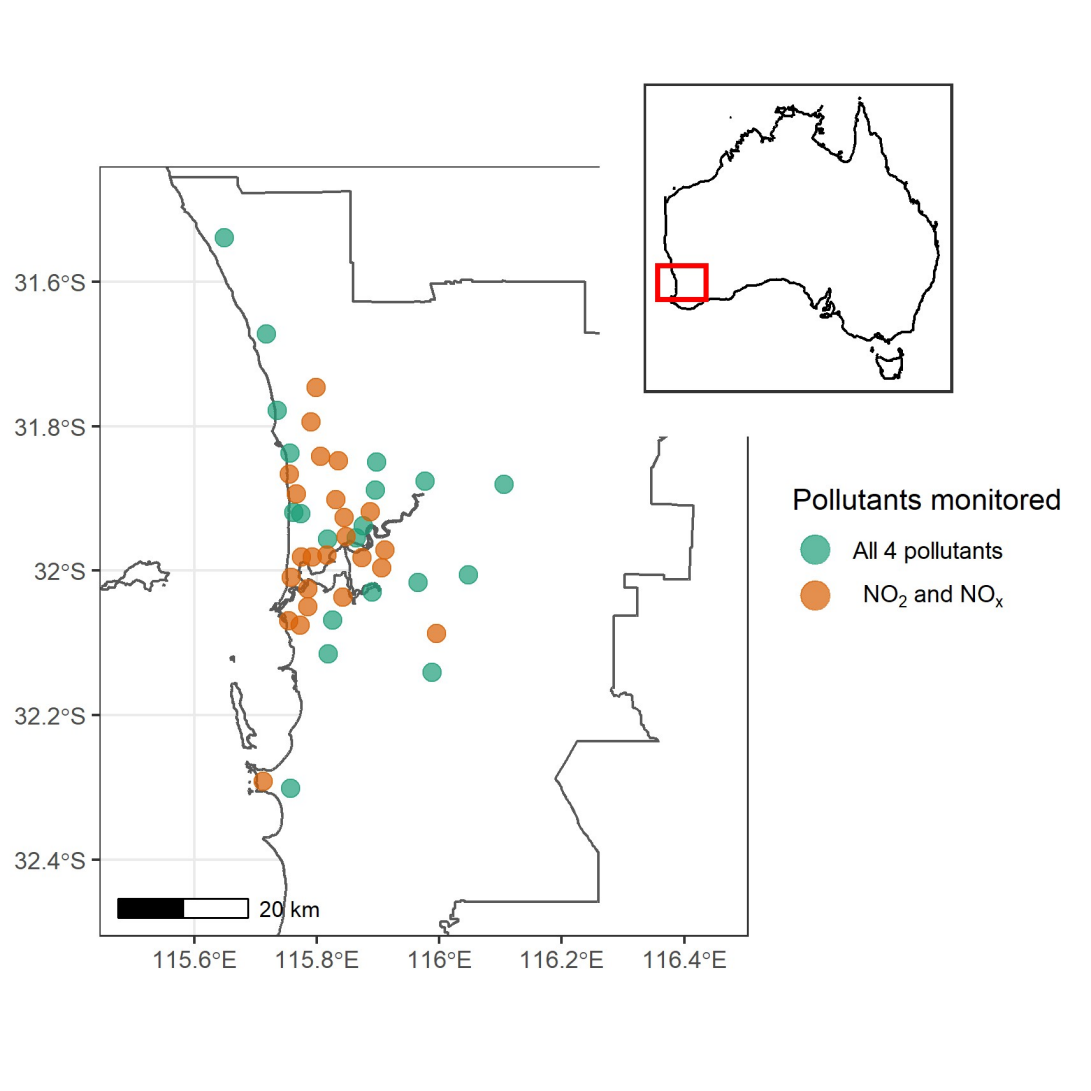
Location of HIMAQs air monitoring sites, Perth, Western Australia, 2012.

Methods for estimating long-term exposure to PM_2.5_, BC, NO_2_, and NO_x_ for participants of the HIMS cohort have been described previously [27, 28]. In brief, Land use regression (LUR) models were developed following the European Study of Cohort for Air Pollution Effects (ESCAPE) protocol (http://www.escapeproject.eu/manuals/). The annual average concentrations of air pollutants were derived from a three-season (summer, autumn, and winter), two-week air monitoring campaign across the metropolitan area of Perth, from January 31 to September 5, 2012, with adjustment for temporal variations. Oxides of nitrogen samples were collected onto Ogawa passive samplers at 43 sites, while PM_2.5_ samples were collected on a Teflon filter using a Harvard Impactor at 20 sites. Reflectance of PM_2.5_ samples was measured using Smoke Stain Reflectometer to obtain PM_2.5_Absorbance concentrations (absorption per unit mass), a measure of black carbon. A map of the study area that shows the locations of sites used to monitor all 4 pollutants (n = 20) as well as which monitors measured only NO_2_ and NO_x_ (n = 23) is presented in Fig 1. The sites were selected to adequately capture the spatial variation of residential addresses for participants involved in the HIMS cohort at the same time as reflect a range of potential air pollution sources including traffic-related emissions, population density, and varying land uses.

Manual stepwise-selection regression procedure was used to select the best predictors of each air pollutant. The performance of LUR models for each air pollutant demonstrated by adjusted R^2^ of both final and validation models were >60%.

Air pollution exposures for the study year corresponding to when the blood sample was taken (2001–2004) were obtained by back extrapolating the predicted concentrations from LUR models, based on annual concentrations sourced from the fixed air monitoring network from the Western Australia Department of Environment Regulation. Back-extrapolation was done by the standardized procedure in the ESCAPE study (http://www.escapeproject.eu/manuals/) and has been detailed in a separate analysis of this same cohort [29].

Data from fixed air monitoring network for black carbon during follow-up period were not available, and so it was not possible to estimate past exposures using the above method. As black carbon concentrations were correlated with NO_2_ (r = 0.7), temporal NO_2_ concentrations were used as proxy for temporal change in BC concentrations. That is measured concentrations of NO_2_, derived from the fixed monitoring network over follow-up period were used to predict changes in concentrations of black carbon.

**Fig 2:**
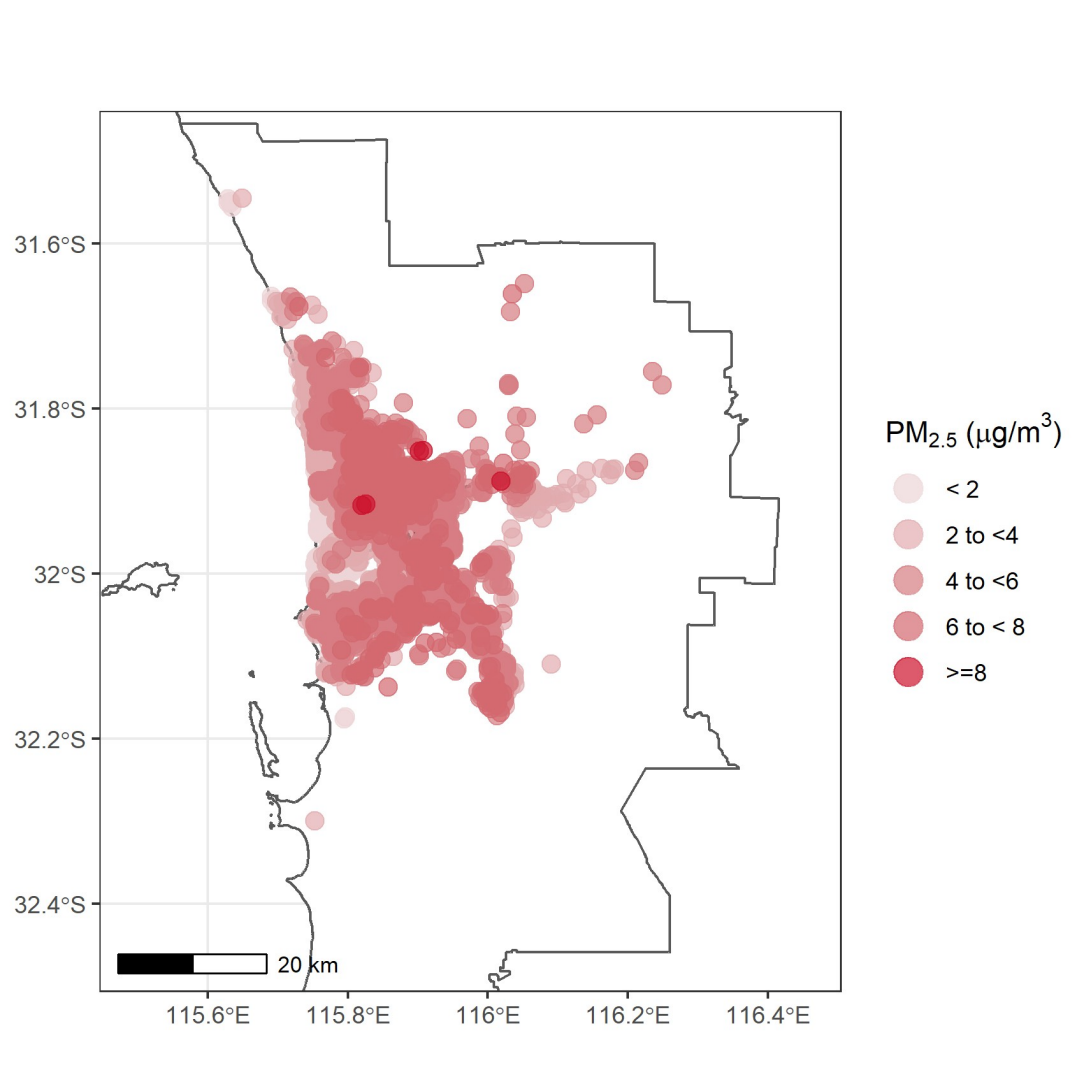
Spatial distribution of modelled PM_2.5_ for HIMS participants (wave II) across metropolitan Perth.

The resulting annual mean air pollutant concentrations between 2001 and 2004 were assigned as long-term exposure for each participant. The predicted annual average concentrations of PM_2.5_, BC, NO_2_, and NO_x_ at participant’s home address were assigned as exposures for participants who were attended the follow-up survey in 2001 (n = 43), 2001 (n = 1,311), 2003 (n = 1,553) and 2004 (n = 1,219), respectively. The spatial distribution of estimated PM_2.5_ exposures for participants included in this analysis is presented in Fig 2.

### Outcome variables

Blood samples were obtained from 4279 men (fasting in 3328 and non-fasting in 921 men). Total cholesterol, high-density lipoprotein (HDL), low-density lipoprotein (LDL), triglycerides, hs-CRP, and tHcy were all measured on the day of blood sampling. Physical measurements were also made, including systolic blood pressure (SBP) and diastolic blood pressure (DBP). At the follow-up survey, blood pressure was measured twice on the upper arm of each participant, after they remained seated for 5 minutes, using an automated sphygmomanometer and recorded to the nearest 2 mmHg. The average of the two measurements was used in all analyses presented here. Summary statistics for each risk factor or biomarker are presented within categories of demographic characteristics in Table 1. Rational for including of tHcy and hs-CRP in this analysis was on the basis that they are markers of inflammation and oxidative stress [30] which are in turn considered as plausible pathways between exposure to ambient air pollution and CVD. Total cholesterol was included as an outcome together with the other lipid fractions to facilitate comparison of findings with other similar studies all of which routinely also included these same outcomes.

**Table 1:** Descriptive statistics of CVD risk factors (2001–2004).

Outcomes (unit)	No. missing	Mean ± SD	Min	Q1	Median	Q3	Max
tHCY (mol/L)	1	13.4 ± 5.6	1.2	10.3	12.4	15.1	147
Hs-CRP (mg/L)	1	3.8 ± 7.3	0.15	1	1.87	3.8	182
SBP (mmHg)	3	146 ± 20.1	86	132	145	158	240
DBP (mmHg)	7	73.8 ± 10.3	1	66	73	81	115
Total Cholesterol (mmol/L)	0	4.9 ± 1	1.9	4.2	4.9	5.5	9
LDL Cholesterol (mmol/L)	2	2.9 ± 0.9	0.1	2.3	2.8	3.4	6.9
Triglycerides (mmol/L)	0	1.4 ± 0.4	0.4	1.1	1.3	1.6	3.7
HDL Cholesterol (mmol/L)	1	1.3 ± 0.8	0.1	0.8	1.2	1.6	10.8

tHcy: total homocysteine; hs-CRP: high sensitivity C-reactive protein; HDL; high-density lipoprotein; LDL: low-density lipoprotein; SBP: systolic blood pressure; DBP: diastolic blood pressure.

### Statistical analysis

Associations between each exposure variable and CVD risk factors (hs-CRP, total homocysteine, total cholesterol, LDL cholesterol, HDL cholesterol, triglycerides, SBP, and DBP) were evaluated using linear regression. Outcome variables were log transformed to normalize distributions and also to limit the influence of extreme values. Associations were presented as percent change for increases per interquartile range width (IQR_w_) increase in exposure which equated to 2.25 μg/m^3^ PM_2.5_, 0.4 x 10^-5^m^-1^ PM_2.5_abs, 6.25 μg/m^3^ NO_2_, and 16.5 μg/m^3^ NO_x_. The convention to report as percent change for increases per IQR_w_ was taken here as this was a metric commonly used in similar studies and compared against in the discussion below. Adjusted mean levels (least square means) of risk factors were also presented by quartiles of each exposure variable.

In order to control for potential confounding, all analyses included smoking (never, former-smokers who had quit ≥10years before the baseline, former-smoker who had quit <10years before the baseline, and current-smokers), education attainment (completed university, completed high school, completed less than five years of high school, and completed some primary school or never attended school), cardiovascular disease history, and body mass index (BMI, in kg/m^2^). This model included a minimum set of a priori defined covariates (referred to as the minimum model) and was used as a sensitivity analysis to compare against the main analysis which included further lifestyle and medication covariates. Specifically, the main model included further adjustment for an area based socio-economic index (SEIFA) [31], a lifestyle score derived as the simple tally of eight prudent lifestyle indicators including diet related behaviors [32], and history of blood pressure and cholesterol lowering medication usage. Although it’s not clear that lifestyle behaviors for this study population would be associated with air pollution exposure, lifestyle is an important predictor of CVD even in old age. Therefore, the purpose of this additional adjustment in the main analysis was to address the issue of confounding more thoroughly including the potential for residual confounding indirectly through socio-economic status.

A number of additional sensitivity analyses were carried out to assess the robustness of results from the main analysis. The linearity of the association between exposure to each air pollutant and outcome was examined by including the exposure variable in a generalized additive model. Influence of outliers was investigated by visual inspection of residual plots and models were re-run after excluding extreme outliers. Assessment of outliers was particularly relevant for analyses involving hs-CRP as the distribution of hs-CRP was very (positivity) skewed. Statistical analyses were performed with STATA software [33] and R [34].

Possible effect modifiers were examined by including interaction terms in the main model. We investigated age (≥ 80 years vs. < 80 years), obesity (BMI ≥ 30 kg/m^2^ vs. < 30 kg/m^2^), education (≥ 10 years versus < 10 years), History of CVD (yes vs. no), and history of diabetes (yes vs. no). Disease history was defined based on linked hospitalization data obtained from the Western Australia Data Linkage System. Hospitalization data records, dating back to 1970, were searched across principal diagnosis codes as well as the next 9 additional diagnoses for each participant for admissions involving CVD (ICD-9 390 to 434; ICD-10-AM I00 to I78) and diabetes (ICD-9 250; ICD-10-AM E10 to E14) respectively.

## Results

### Study population

Among the 4249 men who participated in the second wave of HIMS and provided a blood sample, participants who lived outside the metropolitan Perth region (n = 132) were excluded from all analyses. A summary of study characteristics for the remaining 4117 eligible participants is presented in Table 2. For the main model, there were an additional 19 men who were excluded due to missing data on the key covariate data. The characteristics of men included in these analyses differed slightly from the characteristics of participants surveyed at baseline [25]. Those resurveyed tended to have higher levels of education (22% versus 16% completed university). At the time of the resurvey, study participants were on average 76.5 years old, had a mean BMI of 26.6 kg/m2, and 5% reported they were current smokers. Based on linked hospitalization data, 2106 (51%) of participants had a history of CVD and 377 (9%) of participants with a history of diabetes. A further break down of disease history revealed almost 30% of participants had been admitted to hospital for a coronary heart disease related event.

**Table 2:** Description of the study population.

Variable	Missing values	All
	N	(N = 4,117)
Age ^a^ (years), mean ± SD		76.5 ± 3.6
% total study population based on year of attendance, n (%)		
2001		43 (0.8)
2002		1,311 (31.8)
2003		1,553 (37.7)
2004		1,219 (29.6)
Smoking status ^b^, n (%)		
Never-smokers		1,382 (33.6)
Former smokers who had quit smoking ≥10years before the baseline		2,287 (55.6)
Former smokers who had quit smoking <10years before the baseline		239 (5.8)
Current-smokers		209 (5.1)
Daily tobacco consumption among current smokers (grams/day), mean ± SD		11.7 ± 9.1
BMI (kg/m^2^), mean ± SD	17	26.6 ± 3.6
Education level, n (%)	2	
Completed university		911 (22.1)
Completed high school		1,068 (25.6)
Completed some high school		1,532 (37.2)
Completed primary school or never attended school		604 (14.7)
Lifestyle score ^c^, mean ± SD	199	4.5 ± 1.4
Treatment for Blood Pressure, n (%)	183	
No		2,690 (68.4)
Yes		1,244 (31.6)
Treatment for Cholesterol, n (%)	183	
No		3,170 (80.6)
Yes		764 (19.4)
CVD History ^d^, n (%)		
Any CVD		2,106 (51.2)
CHD		1,187 (28.8)
		73 (1.8)
Diabetes History ^d^, n (%)		
No		3,740 (90.8)
Yes		377 (9.2)

^a)^ Age at time of the wave II survey, determined as the differences between the date of survey and date of birth.

^b)^ Smoking status determined based on data collected during the wave II survey.

^c)^ Lifestyle score was derived as the sum of 8 individual lifestyle factors to produce a total out of 8.

^d)^ Disease history defined on the basis of the principal diagnosis code as well as the next 9 additional diagnoses for each participant using linked hospitalization data. Disease history categories are not mutually exclusive.

BMI: body mass index; CVD = Cardiovascular Disease; CHD = Coronary Heart Disease; PAD = Peripheral Arterial Disease.

### Long-term air pollution

Descriptive statistics of annual concentrations of air pollutants at participants’ residences during the year that the blood sample was collected are presented in Table 3. Modelled long-term exposure to PM_2.5_ was on average 4.5 ug/m^3^ (± 1.6). The maximum modelled concentrations for PM_2.5_ and NO_2_ within the study area during 2001–04 were slightly above the National Environment Protection Measure for Ambient Air Quality (Air NEPM) [35]. Correlations between estimated air pollutants were high between NO_2_ and NO_x_ (r = 0.98), moderate between BC and NO_2_ and NO_x_ concentrations (r = 0.7) low to moderate between PM_2.5_ and NO_2_ and NO_x_ (r<0.4).

**Table 3:** Descriptive statistics of residential air pollutant exposure as long term annual concentrations (2001–2004).

Pollutants (unit)	Mean ± SD	Min	Q1	Median	Q3	Max
PM_2.5_ (μg/m^3^)	4.5 ± 1.6	0.002	3.4	4.7	5.7	9.4
BC (10^-5^m^-1^)	1.0 ± 0.3	0.1	0.8	1.0	1.2	2.0
NO_2_ (μg/m^3^)	14.3 ± 4.5	0.2	11.1	14.0	17.4	30.6
NO_x_ (μg/m^3^)	32.4 ± 12.3	0.1	23.7	31.5	40.1	80.5

PM2.5: particulate matter ≤2.5μm in diameter; BC: black carbon; NO_2_: nitrogen dioxide; NOx: nitrogen oxides.

### Regression analyses

**Fig 3:**
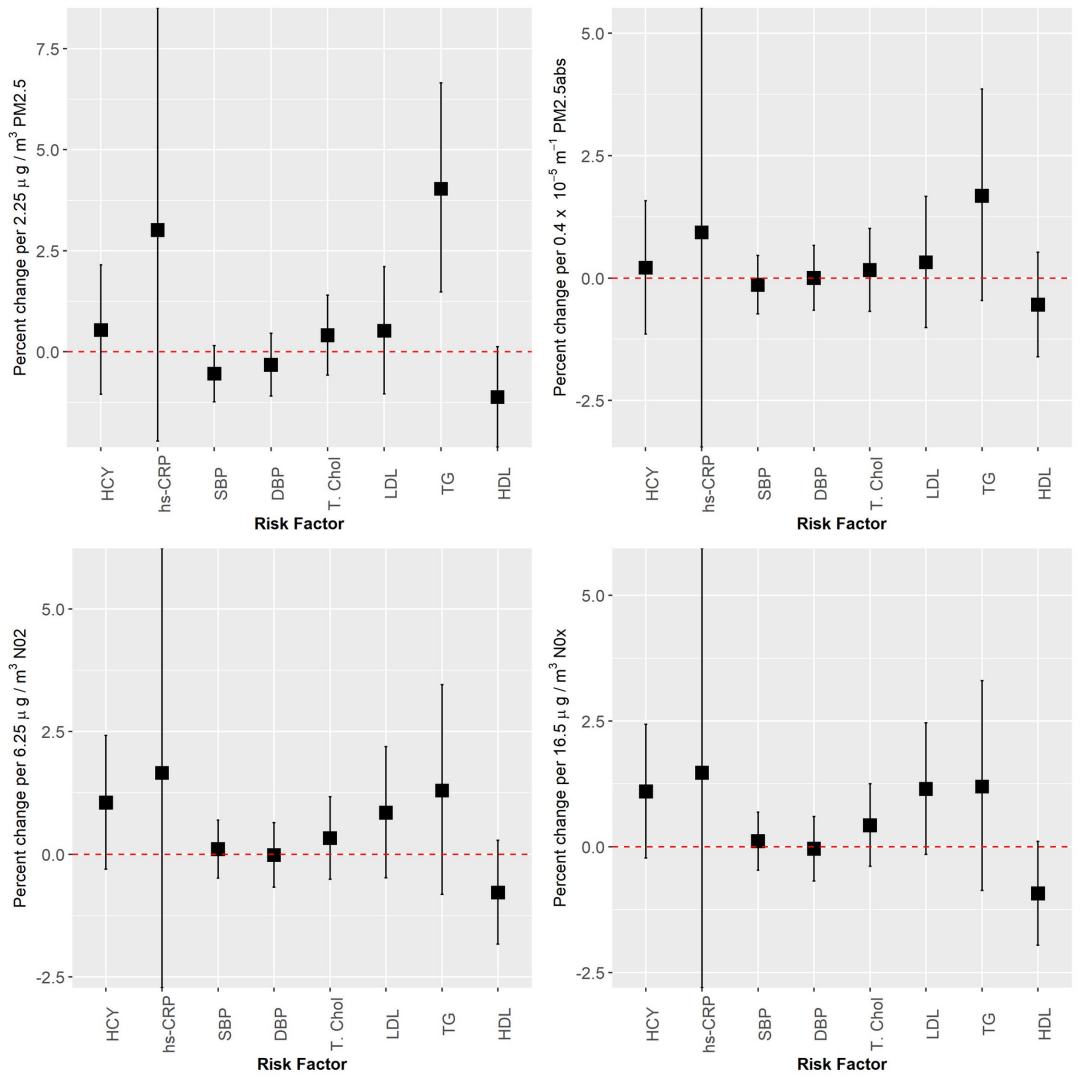
Effect sizes of the association between long-term exposure to air pollution and risk factors for cardiovascular disease, per IQR_w_ increase in air pollutant exposure under the main model.

**Fig 4:**
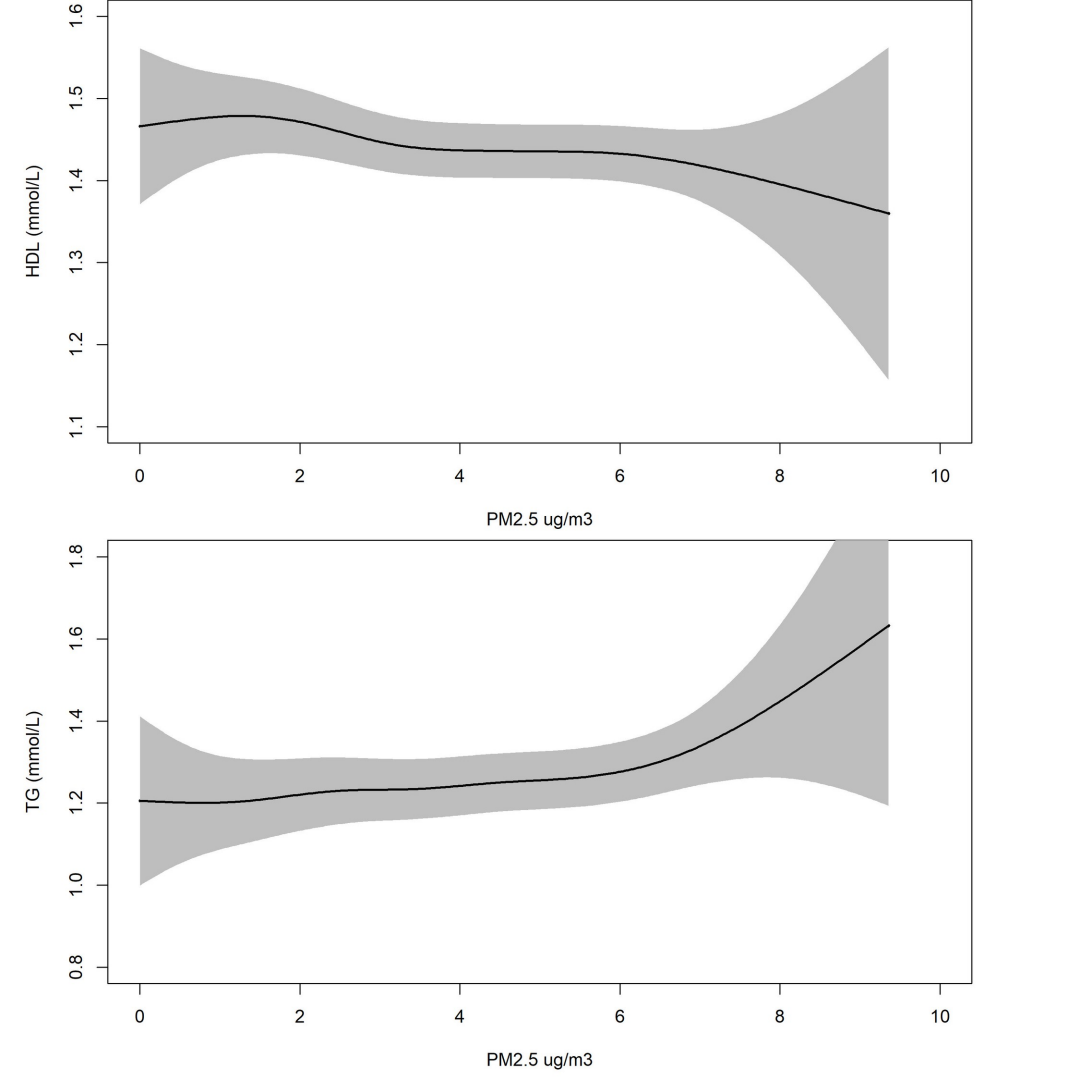
**Smoothed association between fine particulate matter (PM**_**25**_**) and HDL cholesterol (top panel) and triglycerides (bottom panel) adjusted for the main confounders.** Grey shade indicate the 95% confidence interval. Smoothed association estimated using a generalized additive model which included the same covariates as for the main model. Association is illustrated as the linear predictor of outcome for a typical cohort member and presented on the original scale of the outcome. That is, expected mean response for an individual with mean age, BMI, SEIFA, and lifestyle scores; never smoker, attended high school, and with no history of cvd or taking treatments to lower blood pressure or cholesterol; Note that the estimated association has the shape (only scaled up/down) for individuals with differing covariate values as the main model does not include interaction terms.

Estimates of the association between each of the four air pollutants and eight risk factors are presented in Table 4 and the results under the main model are also presented graphically in Fig 3. A linear model was adequate for all outcomes investigated. To illustrate this, results from the generalized additive model, based on the same covariates as for the main model, applied to HDL cholesterol and triglycerides are presented in Fig 4. Smoothed associations were observed to be increasing for PM_25_ and triglycerides and decreasing for PM_25_ and HDL cholesterol with no indication of a threshold apparent in either outcome. Note that Fig 4 presents the association based on the linear predictor for a typical cohort member on the original scale of the outcome. That is, the expected mean response for an individual with average age, BMI, SEIFA, and lifestyle scores; never smoker, attended high school, and with no history of either CVD or taking treatments to lower blood pressure or cholesterol. Note that the estimated association has the same shape (only scaled up/down) for individuals with differing covariate values as the main model does not include interaction terms. For all exposures, and IQR_w_ increase in air pollutant concentration was positivity associated with an increase in hs-CRP and total homocysteine but the associations were not significant. Similarly, positive non-significant associations were observed across all analyses involving Total and LDL cholesterol. Associations between exposures and both blood pressure measures were not significant and with narrow confidence intervals. Our strongest findings were seen for PM_2.5_ with HDL cholesterol and triglycerides where an IQR_w_ increase was associated with a 4.0% increase in triglycerides (95% CI = 1.5 to 6.6%) and a 1.1% decrease in HDL cholesterol (95% CI = -2.4 to 0.1%).

**Table 4:** Effect sizes and 95% CI of the association between long-term exposure to air pollution and risk factors for cardiovascular disease, per IQR_w_ increase in air pollutant exposure.

		Percent change per IQR_w_^a^ (95% CI)			
		PM_2.5_	PM_2.5_abs	NO_2_	NO_x_
HCY	minimum model ^b^	1.2, [-0.2 to 2.6]	0.6, [-0.8 to 1.9]	1.4, [0.1 to 2.8]	1.4, [0.1 to 2.8]
	main model ^c^	0.5, [-1.0 to 2.2]	0.2, [-1.1 to 1.6]	1.1, [-0.3 to 2.4]	1.1, [-0.2 to 2.4]
hs-CRP	minimum model ^b^	3.3, [-1.2 to 8.0]	1.4, [-2.9 to 5.9]	2.1, [-2.2 to 6.6]	1.7, [-2.5 to 6.1]
	main model ^c^	3.0, [-2.2 to 8.5]	0.9, [-3.5 to 5.5]	1.7, [-2.7 to 6.2]	1.5, [-2.8 to 5.9]
DBP	minimum model ^b^	-0.2, [-0.9 to 0.4]	0.1, [-0.6 to 0.7]	0.1, [-0.6 to 0.7]	0.0, [-0.6 to 0.6]
	main model ^c^	-0.3, [-1.1 to 0.5]	0.0, [-0.7 to 0.7]	0.0, [-0.7 to 0.6]	0.0, [-0.7 to 0.6]
SBP	minimum model ^b^	-0.1, [-0.7 to 0.5]	0.0, [-0.6 to 0.6]	0.2, [-0.4 to 0.7]	0.2, [-0.4 to 0.7]
	main model ^c^	-0.5, [-1.2 to 0.2]	-0.1, [-0.7 to 0.5]	0.1, [-0.5 to 0.7]	0.1, [-0.5 to 0.7]
T. Chol	minimum model ^b^	0.1, [-0.7 to 1.0]	0.1, [-0.7 to 1.0]	0.3, [-0.5 to 1.1]	0.4, [-0.4 to 1.2]
	main model ^c^	0.4, [-0.6 to 1.4]	0.2, [-0.7 to 1.0]	0.3, [-0.5 to 1.2]	0.4, [-0.4 to 1.3]
LDL	minimum model ^b^	-0.1, [-1.5 to 1.2]	0.2, [-1.1 to 1.5]	0.8, [-0.5 to 2.1]	1.0, [-0.2 to 2.3]
	main model ^c^	0.5, [-1.0 to 2.1]	0.3, [-1.0 to 1.7]	0.8, [-0.5 to 2.2]	1.1, [-0.2 to 2.5]
Triglycerides	minimum model ^b^	5.2, [3.0 to 7.5]	2.3, [0.2 to 4.4]	1.4, [-0.7 to 3.5]	1.2, [-0.8 to 3.3]
	main model ^c^	4.0, [1.5 to 6.6]	1.7, [-0.5 to 3.9]	1.3, [-0.8 to 3.5]	1.2, [-0.9 to 3.3]
HDL	minimum model ^b^	-1.8, [-2.9 to -0.8]	-0.8, [-1.8 to 0.3]	-0.9, [-2.0 to 0.1]	-1.0, [-2.0 to 0.0]
	main model ^c^	-1.1, [-2.4 to 0.1]	-0.5, [-1.6 to 0.5]	-0.8, [-1.8 to 0.3]	-0.9, [-2.0 to 0.1]

HCY: homocysteine; hs-CRP: high sensitivity C-reactive protein; IQR: interquartile range; NO2: nitrogen dioxide; NOx: nitrogen oxides; PM_2.5_: particulate matter with aerodynamic diameter < 2.5 μm; BC: black carbon (measured as absorbance of PM_2.5_.

^a^ effect estimate is based on IQR_w_ increase which corresponded to 2.25 μg/m^3^ PM_2.5_, 0.4 10^-5^m^-1^ PM_2.5_abs, 6.25 μg/m^3^ NO_2_, and 16.5 μg/m^3^NO_x_.

^b^ minimum model for each risk factor adjusted for age, smoking history (never-smokers, former-smokers who had quit ≥10 years, former-smokers who had quit <10years, current-smokers), smoking intensity among current smokers (# tobacco products /day), education level (completed university, completed high school, completed less than five years of high schools, and completed some primary school or never attended school), BMI, and history of cvd.

^c^ main model additionally adjusted for SEIFA score, prudence score, and treatment for either elevated blood pressure or cholesterol.

### Effect modification

**Fig 5:**
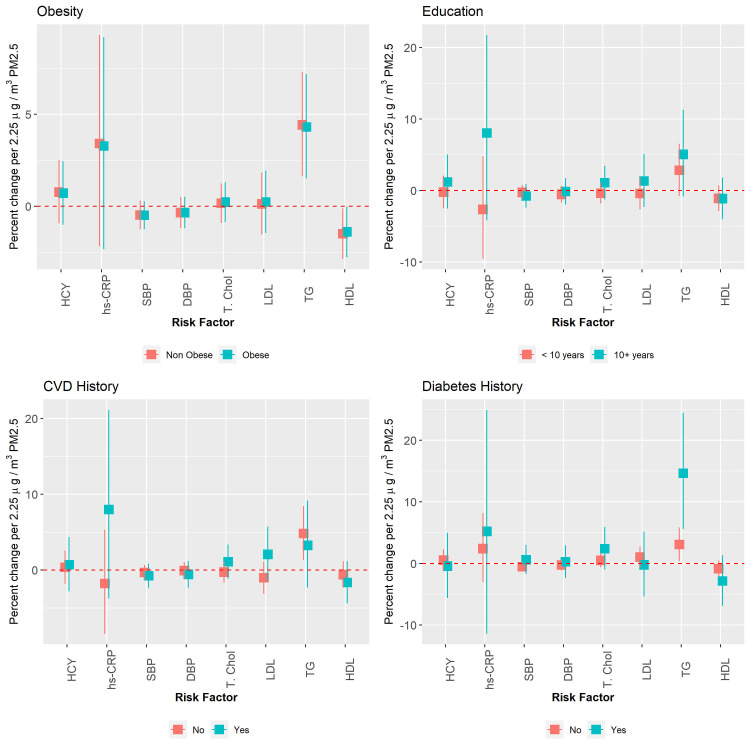
Effect modification of the association between long-term exposure to air pollution and risk factors for cardiovascular disease, per IQR_w_ increase in PM_2.5_ exposure under the main model.

Effect modifications of the association between each of the eight risk factors and exposure to PM_2.5_ are shown in Fig 5 for selected sub populations. The pattern for the other three pollutants examined was very similar with regard to direction and magnitude of association. Results for these analyses are presented in the (S1–S3 Figs). Estimated effect sizes tended to be higher for all risk factors in participants with 10 or more years education. Effect sizes were also larger in magnitude for both biomarkers of inflammation within the sub-group of participants with a history of CVD or diabetes. The largest effect modification was observed for triglycerides in those participants with a history of diabetes and which was statistically significant for PM_2.5_ (p = 0.007) and BC (p = 0.015) but not significant for NO_2_ and NO_x_. No clear differences were observed when the cohort was split by older age (80+ versus <80 years) or BMI (30+ versus < 30 kg/m2). With the exception of blood pressure, confidence intervals for estimates of effect modification were generally wide. Apart from history of diabetes, all other tests of interactions were not significant.

### Sensitivity analyses

In comparison with the main model, the effect sizes changed only marginally when applying the model with the minimum set of covariates. The general pattern with regard to both direction and magnitude of association was very similar and the confidence interval around the effect size for triglycerides clearly excluded zero regardless of which analysis was carried out. Our results for the hs-CRP remained non-significant and with wide confidence intervals after the exclusion of participants having hs-CRP values greater than 20 mg/L (N = 106).

## Discussion

This study investigated the association between long-term exposure to ambient air pollutants and established risk factors for CVD and blood markers for inflammation in a population of older men living in Perth, Western Australia, a city with low ambient air pollution concentrations. We found that higher levels of long-term fine particulate matter were associated with greater levels of serum triglycerides and decreased HDL cholesterol. Although not significant, the results showed a similar trend when analyzed against BC, NO_2_ and NO_x_. No association was found between air pollutants with blood pressure or total cholesterol, and a non-significant but positive association was observed with hs-CRP and total plasma homocysteine.

There are several potential limitations of this study that need to be considered here. The main limitation is that risk factors and biomarkers were only measured at one point in time and therefore the analysis is cross-sectional. While this prevents us from assessing the effect between exposure and outcome over time, we note that spatial variations within the study region did not markedly change over time. Our air pollutant model provided reasonably accurate estimates of long-term exposure, however, measurement error in our exposure estimates cannot be ruled out from our analyses. That said, measurement error is likely to be independent of the outcomes studied here and would be expected to bias estimates of association toward the null. Additionally, there is a possibility of residual confounding due to important covariates either unmeasured or measured with error. Finally, while measures of pollutants other than PM were available, the role of ultrafine particles and inflammation could not be studied here and this is a growing area of interest [23]. For the above reasons, associations from this study should be interpreted with caution.

The main strength of this analysis is that the HIMS cohort is an ideal cohort to investigate possible links between air pollution and cardiovascular disease primarily because older people are more susceptible to the effects of air pollution. Furthermore, there is evidence that these effects in the elderly are seen at lower concentrations of exposure [36, 37]. A wide range of data from the HIMS cohort was available including well measured demographic and lifestyle factors allowing for a thorough investigation of confounders and potential effect modifiers. The LUR models used to predict annual air pollutant concentrations were developed following the well-documented ESCAPE protocol which has been widely implemented and validated for PM_2.5_ at the city scale. Furthermore, additional sensitivity analyses showed similar estimates of effect size therefore supporting robustness of our results.

The findings presented here are consistent with what has been reported in several large health surveys [6, 7] all suggesting a positive association between chronic PM exposure and increased serum concentration of triglycerides. Further evidence of an association comes from meta-analysis of population surveys where triglycerides levels are shown to be consistently higher in urban areas compared to rural areas [38]. This is of interest as ambient air pollution affects people living in built environments worldwide and may therefore contribute to such differences observed between blood lipid levels. The result suggesting PM exposure may have a detrimental effect on HDL cholesterol is also consistent with other reported findings [9] and which may help explain the association between air pollution and increased CVD risk. One of the most intriguing findings of this study was the apparent effect modification by diabetes history. The link between diabetes and dyslipidemia has been reviewed recently [39] and highlighted that the main lipoprotein abnormalities of patients with type 2 diabetes are increased triglycerides and reduced HDL cholesterol [40, 41]. An increased association between PM and lipids among patients with diabetes has been reported previously [42]. In our study, these effect sizes appeared to be somewhat larger, more specific to triglycerides, and persisted after adjustment for multiple potential confounders. This study population represents a subgroup considered to be more susceptible to ambient air pollution and therefore, if valid, would be useful when assessing generalizability across different settings.

There is some evidence that elevated levels of hs-CRP are associated with increased risk of myocardial infarction [43]. However, epidemiological studies on the link between air pollution and inflammatory biomarkers have reported mixed findings with a review concluding further epidemiologic studies are needed to better quantify the magnitude of hs-CRP level changes in response to PM [44]. A number of studies have found an association between short-term elevations in ambient PM and increased levels of hs-CRP, but some of these were small [45, 46] or conducted only on individuals with pre-existing heart disease [47]. It has been noted that CRP assays may have more limited clinical utility due to unacceptably large intraindividual variation [48, 49] and even minor elevations in hs-CRP have been associated with many conditions that are not inflammatory [50]. The distribution of hs-CRP data available from HIMS and analyzed here was also markedly skewed causing very wide confidence intervals around our estimates. Although non-significant, it was nevertheless interesting to note that PM_2.5_ was positively associated with hs-CRP in our study with the highest associations found in participants with a disease history of CVD or diabetes.

Substantial evidence from animal studies supports the hypothesis that PM exposure is linked to CVD through oxidative stress and systemic inflammation [30, 51]. In turn, mechanisms by which chronic inflammation then contribute to an increased risk of atherosclerosis are multifactorial and include altered lipid metabolism and a reduction in the anti-inflammatory capacity of HDL [52]. High serum triglycerides are considered to be a marker for raised triglyceride-rich lipoproteins such as very low density lipoprotein (usually referred to as remnant cholesterol) which is more likely a cause of atherosclerosis than triglycerides [53]. In light of this, it is also worth reviewing studies involving patients with chronic inflammatory conditions, e.g. rheumatoid arthritis, which are associated with increased risk of CVD [54]. Many of these studies have assessed lipid levels in patients with such conditions [55]. Of note, the most common findings are that elevated serum triglycerides and reduced HDL have been observed in these patients when compared to control patients [56–59]. The finding of elevated triglycerides in patients with chronic inflammatory conditions is thought to be due to an increase in hepatic production and secretion and a decrease in the clearance of remnant cholesterol [52].

The findings presented here adds further weight to the role that lipids may have in explaining the effect of long-term exposure to PM on CVD and warrants further research into what the underlying mechanisms might be. If these results were confirmed, implications for clinicians and public health researchers would be raised as to whether there should be more aggressive screening and use of statins in polluted cities if pollution cannot be easily reversed. Statins have been shown to reduce lung inflammation in patients with COPD by promoting clearance of PM from lung tissue [60–62] and therefore statins might also offer a similar mode of action in CVD.

## Conclusions

The analysis of data from this large study population of older men living in a city with relatively low ambient air pollution, suggest that greater exposure to PM_2.5_ is associated, in a dose-response manner, with greater levels of circulating triglycerides and decreased HDL cholesterol. Our results help fill a research gap as studies on the role of air pollution and CVD risk factors are still scarce as are data from lower pollution setting, and research on mechanisms through which air pollution affects CVD remains inconclusive. These results may be useful for researchers looking for biomarkers that might help identify vulnerable subgroups. The findings may also have important implications as well as early interventions which aim to improve air quality and reduce disease burden even for populations exposed to relatively low levels of air pollution.

## Supporting information

S1 Fig Effect modification of the association between long-term exposure to air pollution and risk factors for cardiovascular disease, per IQRw increase in PM2.5abs exposure under the main model. (TIF)

S2 Fig Effect modification of the association between long-term exposure to air pollution and risk factors for cardiovascular disease, per IQRw increase in NO2 exposure under the main model. (TIF)

S3 Fig Effect modification of the association between long-term exposure to air pollution and risk factors for cardiovascular disease, per IQRw increase in NOx exposure under the main model. (TIF)
