# Supplementary figures and images for: Long-term exposure to outdoor air pollution and risk factors for cardiovascular disease within a cohort of older men in Perth
Source: PLoS One. 2021 Mar 29;16(3):e0248931. doi: 10.1371/journal.pone.0248931 (PMC8006998; doi:10.1371/journal.pone.0248931)

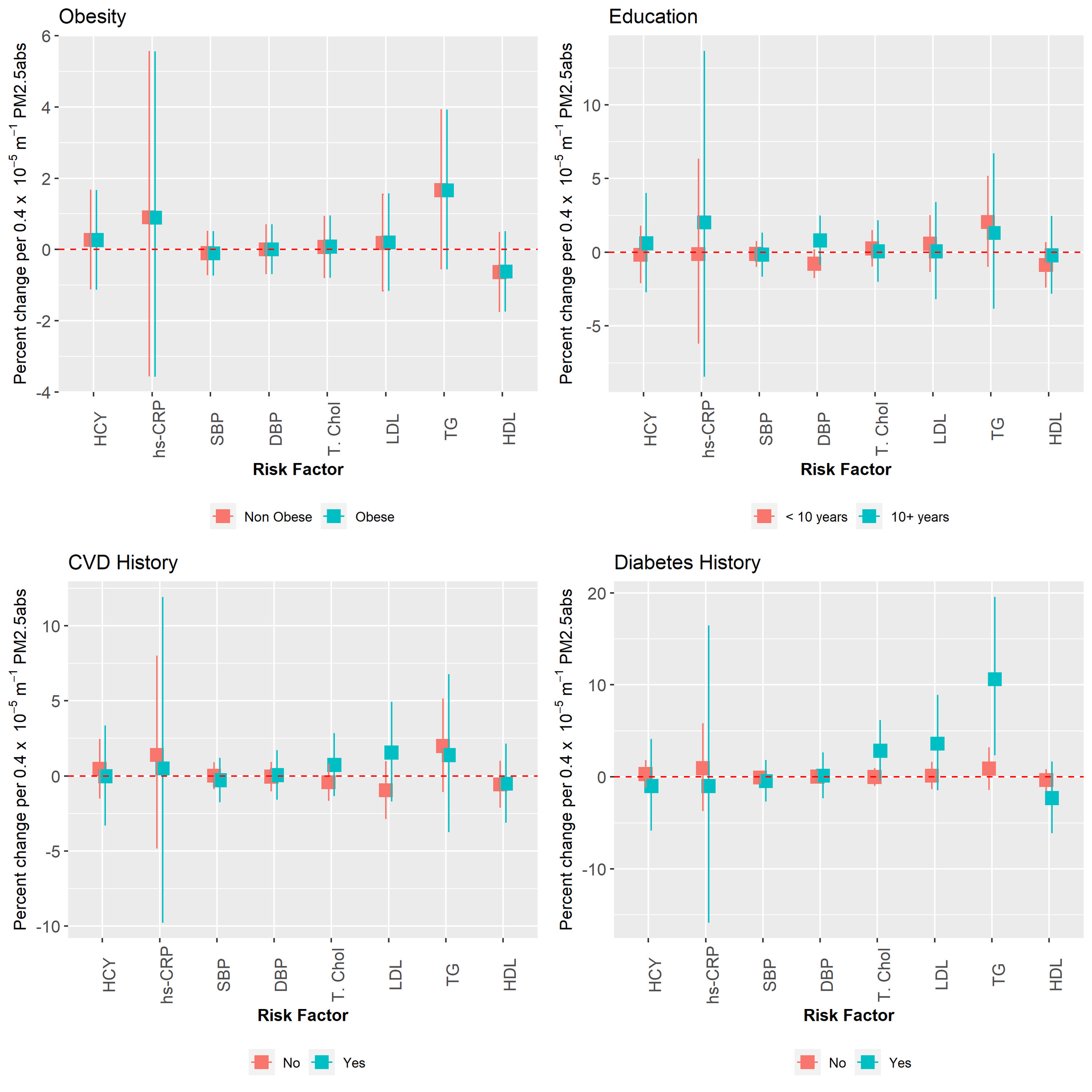

Supplement: S1 Fig — (TIF) [file pone.0248931.s001.tif]

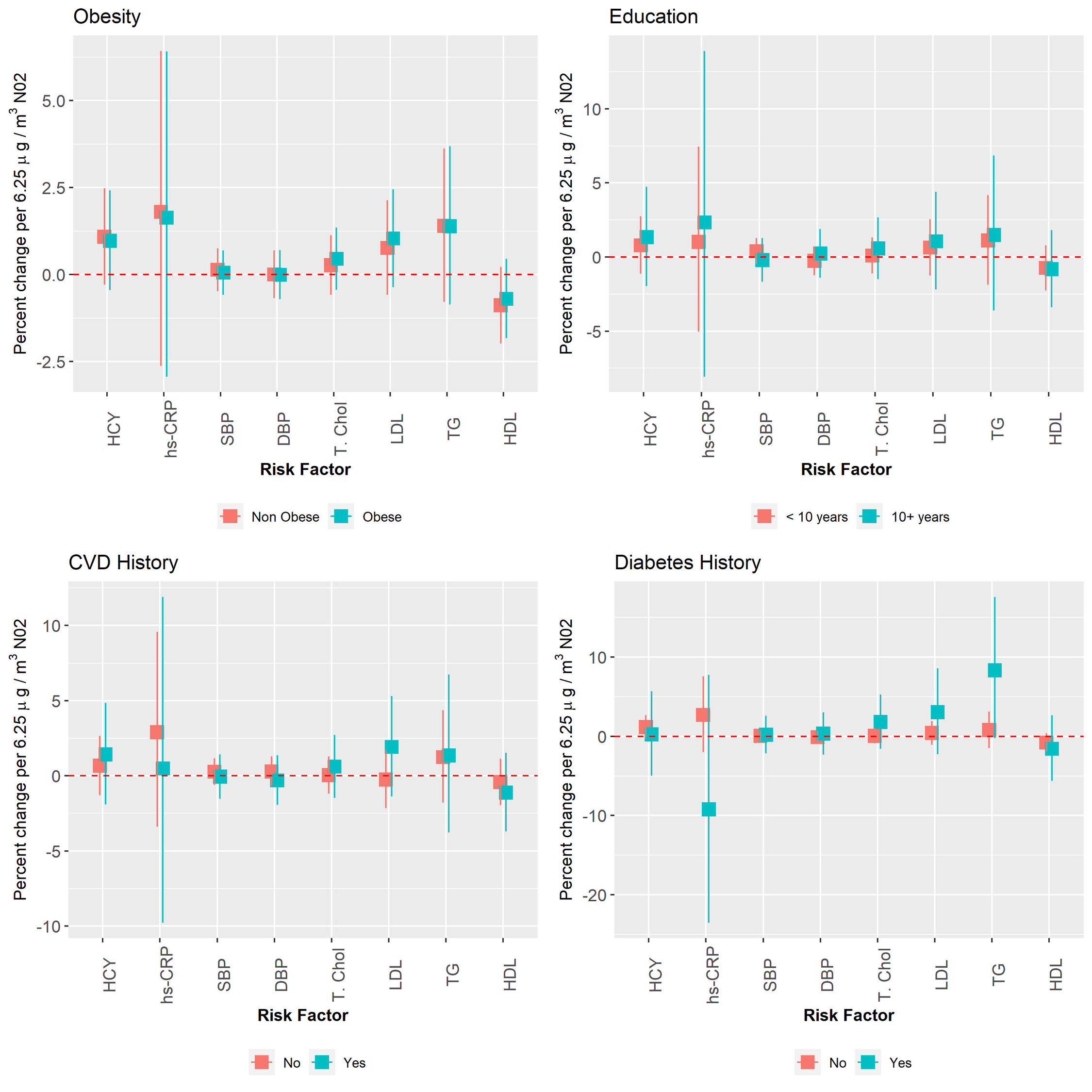

Supplement: S2 Fig — (TIF) [file pone.0248931.s002.tif]

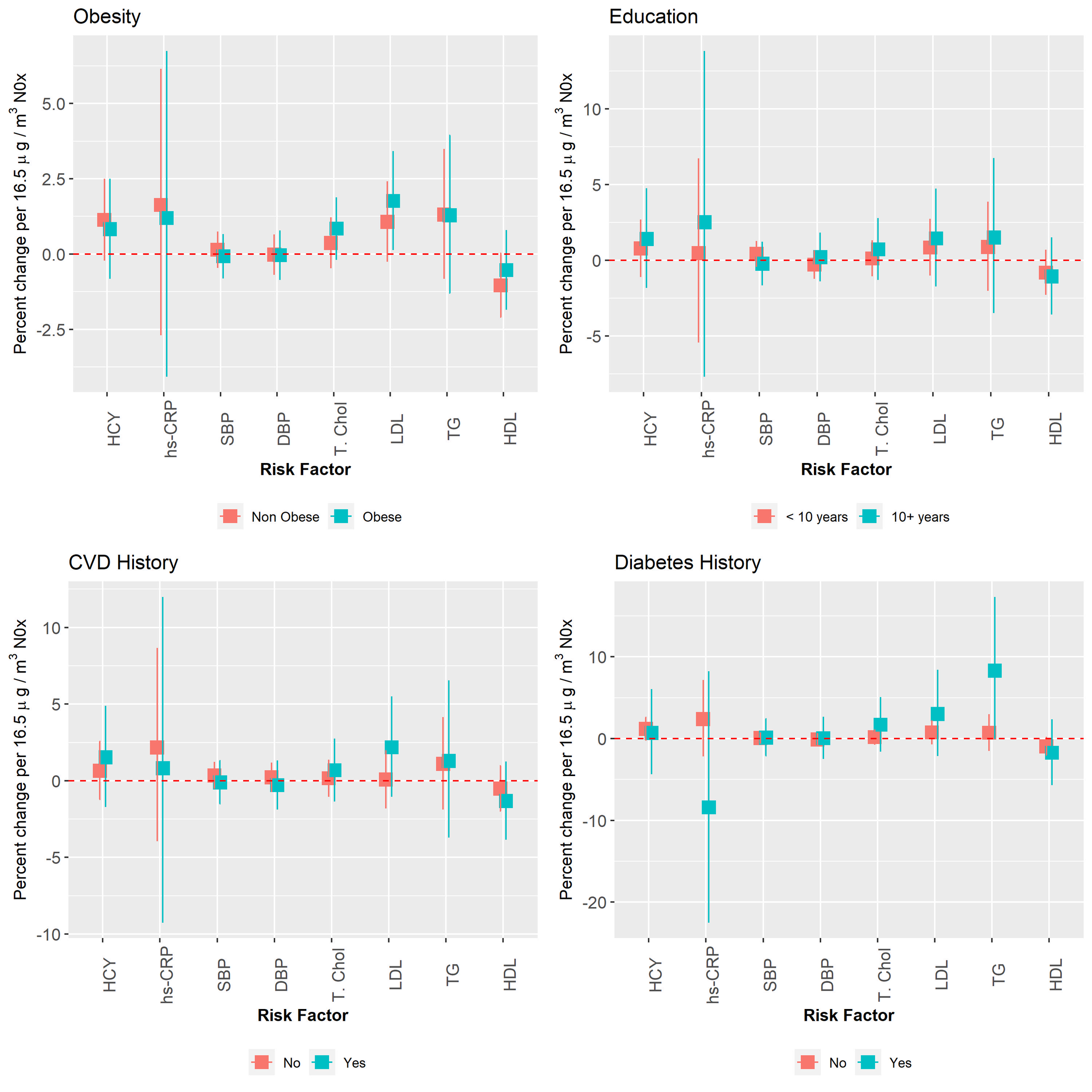

Supplement: S3 Fig — (TIF) [file pone.0248931.s003.tif]
